# Knowledge, Attitude, Behavior, and Practices Regarding HIV, Viral Hepatitis, and Sexually Transmitted Infections Among Migrants From Sub-Saharan Africa Living in Germany: A Multicenter Survey Protocol

**DOI:** 10.2196/resprot.6833

**Published:** 2017-05-02

**Authors:** Claudia Santos-Hövener, Carmen Koschollek, Anna Kuehne, Adama Thorlie, Viviane Bremer

**Affiliations:** ^1^ Robert Koch Institute Department for Infectious Disease Epidemiology Berlin Germany

**Keywords:** KABP survey, HIV, viral hepatitis, STI, migrants from sub-Saharan Africa, community-based participatory health research

## Abstract

**Background:**

Migration has an impact on the epidemiology of viral hepatitis B and C (HEP) and HIV in Germany; migrants from sub-Saharan Africa (MisSA) in Germany are disproportionally affected by HIV. In the last 10 years, a total of 10%-15% of all newly diagnosed HIV cases were among MisSA; 20%-30% of them acquired HIV in Germany. Prevalence of HEP among MisSA in Germany is unknown, but Western Africa, from where most MisSA in Germany originate, reports the highest prevalence of hepatitis B worldwide. There is limited information on knowledge, attitudes, behaviors, and practices (KABP) regarding HIV, HEP, and sexually transmitted infections (STIs), as MisSA are not reached with surveys targeting the general population.

**Objective:**

Our objective was to determine the HIV, HEP, and STI information and prevention needs of MisSA in Germany.

**Methods:**

We conducted a multicenter, cross-sectional, KABP survey regarding HIV, HEP, and STIs among MisSA living in Germany using convenience sampling. The study design was developed as a community-based participatory health research (CBPHR) project; HIV/STI-prevention specialists, key persons from MisSA communities, and HIV/STI researchers were involved in all steps of the research process. Trained peer researchers recruited participants in six study cities. Potential modes of survey administration were interview or self-completion, and the questionnaire was available in English, French, and German. Questions on knowledge about HIV, HEP, and STIs were presented as true statements; participants were asked if they had known the information before. Focus groups with MisSA were conducted to interpret results. Data collection took place from October 2014 to November 2016.

**Results:**

Recruitment by peer researchers concluded with 3040 eligible participants. Data collection was completed in November 2016. We are currently analyzing the quantitative data and qualitative data from focus groups. We are conducting working group meetings to discuss the results in the respective study cities and to evaluate the application of participatory health research in epidemiological studies. First results are expected by the end of 2017.

**Conclusions:**

Working with peer researchers to collect data allowed accessibility to a diverse sample of MisSA and, particularly, allowed us to reach vulnerable subgroups, such as MisSA without legal status. The ability to access hard-to-reach groups is one of the big advantages of CBPHR. The active inclusion of the persons under study in the design of the study resulted in higher acceptance and ownership of the research project in the target community; this ultimately lead to better quality of collected data. Furthermore, the participation of MisSA in the development of study design and data collection assures a better understanding of the interests, needs, and living conditions of this group.

## Introduction

### HIV, Sexually Transmitted Infections, and Hepatitis B and C Among Migrants From Sub-Saharan Africa Residing in Germany

Research shows the impact of migration on the epidemiology of hepatitis B and C (HEP) and HIV in Western Europe [1,2].

#### HIV

Worldwide, an estimated 36.7 million people (range 34.0-39.8 million) were living with HIV at the end of 2015 and 2.1 million people (range 1.8-2.4 million) acquired HIV in 2015. Sub-Saharan Africa is the region mostly affected by HIV, with 25.6 million people (range 23.1-28.5 million) living with HIV in 2015 [3]. In addition, sub-Saharan Africa accounts for two-thirds of new HIV infections worldwide [3].

In Europe, migrants accounted for 40% of newly reported HIV diagnoses and most of these cases were among migrants from sub-Saharan Africa (MisSA) [4]. A total of 12 countries reported to the European Centre for Disease Prevention and Control (ECDC) that the majority of HIV cases with heterosexual transmission originated from high-prevalence countries [4].

In Germany, men who have sex with men are most affected by the HIV epidemic [5,6]. However, heterosexual contacts (HET) have always been the second-most common mode of transmission and have been increasing since 2012 [7]. Between 9% (2012) and 14% (2014) of all new HIV diagnoses in Germany were among persons originating from sub-Saharan Africa. Since 2013, there has been an increase in diagnosed HIV cases, particularly among female MisSA [6-8]. A total of 75% of all newly diagnosed HET cases were people from countries of origin other than Germany; between 40%-50% annually are MisSA [7].

There is no accurate estimate on the number of MisSA living in Germany because population statistics include neither MisSA with German nationality nor those without legal status. The estimated number of MisSA with sub-Saharan nationality was approximately 200,000 in 2013 [9]. Even though this number might underestimate the number of MisSA, it highlights that in comparison to other migrant groups, this population is small and disproportionately affected by HIV. At the beginning of the HIV epidemic in Germany, epidemiologists generally assumed that MisSA acquire their HIV infection in their country of origin. However, current surveillance data has shown that the proportion of HIV infections acquired in Germany varied from 15%-28% in 2009-2014 [10]. This proportion might be underestimated: a UK study showed that the estimated proportion of MisSA who acquired infection within the United Kingdom—based on CD4 cell counts—was three times higher than the figures resulting from clinicians’ reports [11].

MisSA with HIV infection are often late presenters [12-14], potentially due to barriers to HIV testing, prevention, counseling, or health care in general [15]. Therefore, the proportion of undiagnosed MisSA might be higher than in other subpopulations affected by HIV [13].

#### Hepatitis B and C

The estimated prevalence of chronic hepatitis B is 240 million and is highest in sub-Saharan Africa and East Asia, where between 5% and 10% of the adult population is chronically infected with hepatitis B. The reported prevalence of chronic hepatitis B in Western sub-Saharan Africa, from where most MisSA in Germany originate, is up to 14% [16].

In Europe, hepatitis B often occurs in migrants from highly endemic countries. In 2011, 53% of the reported hepatitis B cases from 18 European countries were imported [3,17]. Recent studies show that migrants in Germany show higher sero-prevalence of hepatitis B (2.1%-3.6%) than the German general population (0.3%) [18,19]. However, these studies did not include sufficient samples of MisSA.

Globally, the prevalence of chronic hepatitis C in 2016 is between 130 and 150 million persons [20]. The estimated prevalence of hepatitis C infections in sub-Saharan countries is 3% [16,21,22]. In Europe, only 17 countries reported data on country of birth or importation status for newly diagnosed hepatitis C cases to the ECDC. Of these 12,111 cases, 8.3% were imported [4]. Migrants in Germany show higher sero-prevalence of hepatitis C (1.1%-1.9%) than the German general population (0.3%) [16,17]. However, these studies did not include sufficient samples of MisSA. In Germany, migration status is not collected for reporting of new diagnoses for hepatitis B and C.

#### Sexually Transmitted Infections

According to the World Health Organization (WHO), there is an estimated annual incidence of 357 million new infections with one of four sexually transmitted infections (STIs): chlamydia, gonorrhea, syphilis, and trichomoniasis [23]. The African region contributed approximately 24% of the worldwide incidence of these four STIs in 2005 [24].

In 2010, the ECDC recorded only 11 countries that reported migration status for gonorrhea cases. In those countries, 11% of cases were in migrants and 50% were in nonmigrants. Studies from Europe show no significant differences between infection rates for gonorrhea and syphilis in migrants and native populations [3]. A total of 23 EU countries reported data on syphilis to the ECDC; of those, nine provided information on migration status. The proportion of migrant cases has remained stable around 8.5% [3].

There are no estimates on the incidence of STIs among MisSA living in Germany. Migration status is captured only in syphilis reporting and only 0.3% of reported syphilis cases originated from sub-Saharan Africa [10].

#### HIV, Hepatitis B and C, and Sexually Transmitted Infection Testing in Germany

Testing for HIV, HEP, and STIs in Germany is offered by the local public health departments (LPHDs), medical professionals (eg, gynecologists and HIV specialists), and other local testing and counseling centers (eg, nongovernmental providers). Special prevention services; counseling; and HIV, HEP, and STI testing services for MisSA are offered by AIDS foundations, other nonprofit organizations focusing on migrant health, and LPHDs. The LPHDs have a legal mandate to reach vulnerable populations with HIV, HEP, and STI prevention services and to offer free, anonymous testing. HIV testing is offered based on a personal risk assessment, whereas STI and HEP testing are only offered by the LPHD if patients are presenting with symptoms. However, LPHDs and nongovernmental organizations report problems in reaching migrant populations and, in particular, reaching MisSA with testing services [25].

#### Social and Structural Barriers to Prevention and Behavioral Surveillance Among Migrants From Sub-Saharan Africa

Studies show that migrants, compared to the German general population, have an increased vulnerability for HIV infection because access to HIV prevention, testing, and care is often limited due to legal, cultural, socioeconomic, or language barriers [3,15,26]. In addition, the migration process itself can increase risk of HIV, HEP, and STI acquisition, as migrants might experience (sexual) trauma, discrimination, and problems with legal status [3,15,27,28]. Moreover, the lack of culturally sensitive services and language/translation capacities to reach different migrant groups, as well as migrants’ lack of knowledge about existing services, might present important barriers to prevention. Other aspects are discrimination and stigmatization from outside migrant communities, as well as stigmatization of people living with HIV within migrant communities [15].

Therefore, in order to assess prevention needs and to tailor prevention to these specific needs in vulnerable migrant groups, the ECDC, the WHO, and the Joint United Nations Programme on HIV/AIDS strongly recommend monitoring of behavioral indicators [29], in addition to routine surveillance. Suggested indicators include access to testing, treatment, prevention, and care; knowledge on HIV and STIs; attitudes toward people living with HIV; and (sexual) protective and risk behavior [29,30].

In Germany, data on knowledge, attitude, behavior, and practice (KABP) of MisSA is scarce, and the population is not sufficiently reachable with surveys addressing the German general population [31]. The level of knowledge on HIV and STIs, as well as protective and risk behaviors of MisSA, have so far only been assessed in studies with small sample sizes (ie, <300 participants) [32,33], whereas HEP has not been addressed in any Germany-based study with MisSA.

### Community-Based Participatory Health Research

Community-based participatory health research (CBPHR) is a research approach that includes members of the group under study in the research process [34]. As seen on page 4 of the handbook by von Unger and Gangarova, CBPHR can be defined as a “collaborative approach to research [that] equitably involves all partners in the research process and recognizes the unique strengths that each brings. CBPHR begins with a research topic of importance to the community with the aim of combining knowledge and action for social change to improve community health and eliminate health disparities” [35].

Therefore, one important goal of CBPHR is social change and ultimately improving the situation of the group under study [34,36,37]. Part of this is a process of mutual learning from each other and the establishment of skills and relationships that promote self-esteem of community members [34-36]. The true involvement of community partners leads to a better understanding of the particular needs of community members, improves access to a particular community, and ultimately results in enhanced data quality in epidemiological research [34,38]. Moreover, this mutual working process is building valuable networks that can be utilized for intervention planning and dissemination of results [35,37,38]. Further benefits of utilizing CBPHR in epidemiological studies are the interpretation of results and development of practical recommendations with experts of the group under study as well as other stakeholders. Ideally, this dialogue will lead to concrete prevention activities that are feasible and accepted in the target population [38]. Thus, implementing CBPHR approaches into a behavioral survey with marginalized populations might result in higher quality of data and give an opportunity to understand the social context of the group under study [34,36,39]. In HIV/STI research with MisSA, CBPHR has been utilized for establishment of research design, survey development, data collection, and dissemination of results [32,33,40-44]. Experience from these studies showed the feasibility of this approach, in particular, regarding participant recruitment through trained members from the respective MisSA communities under study (peer researchers) [32,33,40-44].

### Objectives of the Study

Our research interest was to determine the specific HIV, HEP, and STI prevention needs of MisSA in Germany.

The specific objectives were as follows: (1) to identify behavioral determinants, risk, and protective factors in regard to these infections; (2) to determine the information needs on these infections; (3) to assess the utilization of existing testing services (ie, HIV, HEP, and STIs); (4) to sensitize MisSA communities for HIV, HEP, STIs, and other sexual health topics; and (5) to develop culturally sensitive recommendations for prevention with MisSA. This cross-sectional study was also intended to promote the inclusion and empowerment of MisSA communities and the establishment of local networks for prevention planning.

## Methods

### Study Design

We conducted a multicenter, cross-sectional, KABP survey regarding HIV, HEP, and STIs among MisSA living in Germany using convenience sampling. The study design was developed as a CBPHR project with persons working in HIV/STI prevention with MisSA, key persons from MisSA communities, and HIV/STI researchers; we conducted a pilot study to test feasibility of the study design in Hamburg before finalizing the study flow. Details on the description of the research process and the pilot study can be found elsewhere [44,45].

The study was funded by the German Ministry of Health and coordinated by the Robert Koch Institute (RKI), the national public health institute with the mandate for infectious disease surveillance and related research. Ethics approval was received as of November 25, 2014, from the ethics committee at the medical university of Charité, Berlin (EA4/105/14). The study protocol was approved by the Commissioner for Data Protection of the RKI without concern as of January 14, 2015.

### Selection of Study Cities

**Figure 1:**
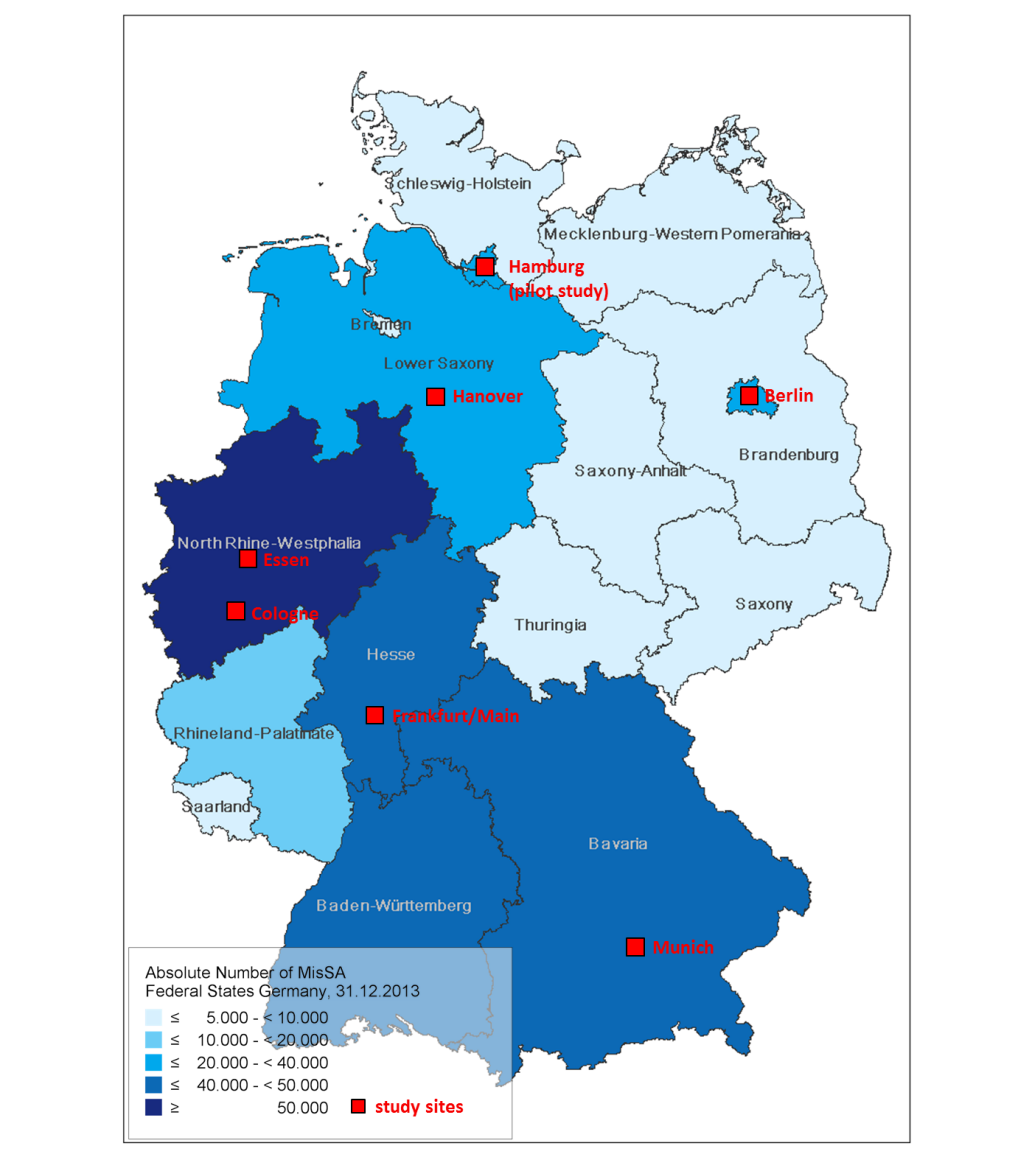
Map of study cities. MisSa: migrants from sub-Saharan Africa.

This study was conducted in six German cities with large MisSA communities (ie, >2000 MisSA residents according to population statistics). Other criteria for selection of study city were as follows: (1) availability of partner organization with well-established contacts to local MisSA communities, (2) willingness of a free and anonymous HIV/STI testing service (eg, the LPHD) to participate, (3) geographic region of city, and (4) no previous research on prevention needs of MisSA communities. After a kick-off meeting in 2014, the following study cities were selected: Munich, Essen, Cologne, Berlin, Frankfurt, and Hanover (see Figure 1). We chose two cities from the Rhine-Ruhr region, a densely populated area of North Rhine-Westphalia with a very big MisSA population. Given the close proximity of cities in this region, MisSA living here are well connected. The structure of the local communities in the respective cities varies, as is the case with Cologne, where the biggest MisSA subpopulation originates from the Democratic Republic of Congo. Essen, on the other hand, has a high number of MisSA from Ghana. By conducting the study in the two big cities in this region, we were able to show the heterogeneity of this area.

In each city, the selected partner organization identified a MisSA community member to work as local study coordinator. The local study coordinator supported the partner organization and the RKI with selecting the peer researchers. In addition, the study coordinator was the liaison communicating between the peer researchers, the RKI, key persons from the community, and study participants.

### Questionnaire Development and Measurements

An expert group consisting of MisSA community members, people working in HIV/STI prevention with migrant groups, representatives from HIV/STI clinics, HIV specialists, and researchers collectively developed the questionnaire used in this study. We included indicators suggested by the ECDC for behavioral surveillance with migrant populations [29]. As a draft, we utilized the survey instrument of a sexual health survey for African communities from the United Kingdom [41], translated the questionnaire into German, and added some questions on HEP and local HIV testing services. African community members conducted cognitive interviews with five migrants from sub-Saharan Africa to determine comprehensibility, recall strategy, issues with sensitivity, and social desirability, as well as understanding the response process [46,47]. Feedback from cognitive interviews was generally positive and only minor modifications were necessary. Before conducting the pilot study in Hamburg, we conducted pretesting of the questionnaire with 35 MisSA and discussed questions with low response rates and comments by participants with the expert group. MisSA community members translated the questionnaire into English and French. Translation was verified by independent native English and French speakers.

After the pilot study, we adapted the questionnaire, pretested it, and adjusted it again together with the expert group. Following data collection in the first study site, Munich, we changed the wording of three questions to improve recallability. We also included two questions on knowledge about female genital mutilation/cutting (FGM/C) suggested by the partner organization in the second study city, Essen.

The final questionnaire consisted of 51 questions and 23 knowledge items and covered the following sections: sociodemography and access to health care; testing behavior with regard to HIV, HEP, and STIs; sexual behavior and risk factors (ie, sexual violence and circumcision/FGM/C); stigmatization; and sources and preferred means of information and knowledge of infections and their transmission. For more details, see Table 1. We used true statements to determine knowledge on transmission and prevention of HIV and HEP (eg, “Hepatitis is a disease of the liver. Did you know this before?”). Participants had the following response options: “I knew this already,” “I was not sure if that was true or not,” “I didn’t know this,” or “I don’t understand this statement.”

**Table 1:** Survey sections and operationalization.

Section	Details and items (numbered)
Administrative information given by peer researcher	Peer ID, mode of administration of questionnaire, date of administration, and city where participant was recruited
Sociodemographic and personal characteristics	Sex, age, country of birth, country of birth of parents, time spent living in Germany, German language proficiency, visiting country of origin, living situation, steady relationship and time spent with steady partner, religious affiliation and participation in religious community life, level of education, sources of income and height of monthly net income, health insurance status, and health care utilization
Knowledge on hepatitis B/C: Use of informing statements “Did you know this before now?”	(1) Hepatitis is a disease of the liver. (2) Hepatitis B and C can be transmitted through blood or used needles. (3) Hepatitis B can be transmitted through sexual contact. (4) Hepatitis B can also be transmitted from mother to child. (5) Chronic hepatitis is a disease that often progresses unnoticed. (6) An untreated hepatitis can lead to cancer. (7) Chronic hepatitis can be treated successfully. (8) There is a vaccination to protect against hepatitis B. (9) There is no vaccination against hepatitis C.
Knowledge on HIV: Use of informing statements “Did you know this before now?”	(10) HIV and AIDS also exist in Germany. (11) AIDS is caused by a virus called HIV. (12) You cannot tell from someone’s appearance whether he or she has HIV or not. (13) There is a test which shows whether someone is HIV positive or not. (14) Africans are NOT deported from Germany just for having HIV. (15) In [study site] you can get tested for HIV anonymously and for free, eg, at the LPHD^a^ (see flyer). (16) People that have sexually transmitted infections have an increased risk of contracting HIV. (17) HIV is not transmitted through kissing or shaking hands. (18) HIV can be transmitted through sexual intercourse. (19) People with HIV have an increased risk of contracting tuberculosis. (20) There is no cure for HIV infection. (21) There are medications that can help people with HIV stay healthy.
Knowledge on STIs^b^ “Have you ever heard of these STIs?”	(22) gonorrhea, (23) syphilis, (24) herpes, (25) genital warts, (26) chlamydia, (27) I do not know any of them
HIV, hepatitis C, and STI testing	Ever tested for hepatitis C or HIV, last time tested and results, country where last HIV test was done, testing for HIV without consent, last time tested for STI and last time diagnosed with STI, preferred HIV- and STI-testing sites
Sexual behavior and risk factors	Age at first sexual intercourse, sexual attraction, sexual activity within the last 12 months, number of male and female sexual partner(s) within the last 12 months, steady sexual partner(s) and origin of steady sexual partner(s), sex with nonsteady sexual partner(s) within the last 12 months, condom use with nonsteady sexual partner(s), condom use at last sexual intercourse, reasons for not using condoms, experience of sexualized violence and country where that happened
Circumcision and female genital mutilation/cutting (FGM/C) Knowledge on FGM/C: Use of informing statements “Did you know this before now?”	Circumcision and female genital mutilation/cutting (28) Did you know that a reconstruction of the female genitalia is possible for persons who have undergone female circumcision? (29) Did you know that the state health insurance in Germany can cover the cost for the reconstruction?
Behavior toward people living with HIV (PLWH)	Reaction and behavior toward PLWH, personal connection to PLWH, ever heard of PLWH being treated badly in community, HIV/AIDS as a topic discussed in community
Self-stated information needs	Relevant topics for prevention, preferred mode of dissemination

^a^LPHD: local public health department.

^b^STI: sexually transmitted infection.

### Participants and Inclusion Criteria

Inclusion criteria for study participants were as follows: (1) 18 years of age or older, (2) born in sub-Saharan Africa or with at least one parent originating from sub-Saharan Africa, (3) currently residing in Germany, and (4) living in or near the respective study city. Peer researchers and local study coordinators were not allowed to take part in the survey.

### Sample Size

The calculated sample size was 3009. Differences in proportions of 10% (ie, 45% vs 55%) between men and women should be detectable with a significance level of .05 and accepting a beta error of .2. Additionally, the smallest group among MisSA (ie, MisSA from Southern Africa), making up about 10% of all MisSA in Germany, should make up at least 8.5% of the sample for stratified analysis, accepting a beta error of .2 and alpha error of .05 (both sided). We were aiming to recruit a minimum of 2550 MisSA in six cities in Germany and were planning to include the sample of MisSA from the pilot study in Hamburg (N=569). We added an extra 110 persons to the calculated sample size to assure the achievement of the total sample size of 3009.

In addition to analysis of the total sample, we wanted to report results of study cities to the respective local partner organizations. Therefore, we decided on a minimum sample size of 350 MisSA per city to enable us to describe proportions of knowledge and sexual behavior as well as demographic characteristics accurately (significance level of .05 and accepting a beta error of .2). To determine the targeted sample size per city, we considered the size of the local MisSA community and the resources of the respective partner organization. Table 2 shows the targeted minimum sample size per city and the registered MisSA [48]. The total targeted sample size in this recruitment period was 2550.

**Table 2:** Expected sample size per study city.

Study city/region		Registered MisSA^a^(population statistics, 2010) [48]	Targeted minimum sample size
**Rhine-Ruhr region**			1000
	Essen^b^	8731	650
	Cologne	5438	350
**Berlin**		12,086	500
	Munich	7970	350
	Frankfurt am Main	5605	350
	Hanover^c^	3377	350
Total			2550

^a^MisSA: migrants from sub-Saharan Africa.

^b^Recruitment in Essen (2229), Mulheim an der Ruhr (909), Oberhausen (911), Duisburg (1571), and Düsseldorf (3111).

^c^Recruitment in the administrative districts Hanover Region (2554), Hildesheim (239), and Brunswick (584).

### Data Collection

Data collection took place from October 2014 to November 2016.

#### Selection and Training of Peer Researchers

The local partner organization selected 10-15 peer researchers for recruitment of study participants. Inclusion criteria for peer researchers were as follows: (1) well-established contacts within their communities, (2) availability during study period and training sessions, (3) interest in sexual health and community work, (4) basic German language skills (for training session), and (5) 18 years of age or older. Peer researchers were recruited by the local partner organization and in agreement with the RKI. They were selected based on the following characteristics: (1) country of birth (based on the biggest subpopulations according to population statistics), (2) gender, (3) age, and (4) spoken languages. In order to recruit a heterogeneous sample of MisSA living in the respective study city, it was important that the biggest subpopulations with regard to countries of birth were adequately represented within the group of peer researchers. Representatives from the RKI, the local study coordinator, and peer researchers from previous study cities conducted a 2-day training session for peer researchers. Topics covered were as follows: (1) study flow, (2) survey administration, (3) ethical aspects of data collection, (4) recruitment strategy (eg, “How do I approach potential participants?”), and (5) basic information on HIV, HEP, and STI transmission and testing. We then conducted a community mapping exercise [33] to identify potential places for recruitment. Here, peer researchers discussed where they meet other MisSA. To explain possible recruitment strategies and their experiences with them, peer researchers from former study cities were invited to the training session. Recruitment of study participants started immediately after the training session and was scheduled to take 10-12 weeks per study city.

#### Recruitment

Trained peer researchers recruited participants through outreach by convenience sampling in locations previously identified though community mapping. The possible modes of questionnaire administration were either face-to-face or telephone interview conducted by the peer researcher or self-completion of the questionnaire by the participant. Verbal informed consent for taking part in the survey was obtained by peer researchers. Peer researchers were asked to preferably offer self-completion of questionnaires to reduce social desirability bias. Study participants received an addressed and stamped return envelope allowing them to mail the questionnaire directly to the RKI. Peer researchers and participants were encouraged to send the questionnaires promptly to the RKI. Participants received a give-away package consisting of a pen with the RKI logo, a shopping cart chip with a map of Africa, a referral to the local partner organization, a condom, and a flyer on the free HIV/STI testing services at the LPHD.

To steer recruitment and make sure the largest African communities were well represented, the RKI provided a weekly summary of the sociodemographic characteristics of the recruited participants to the local study coordinator. The following indicators were included: mode of administration, language of questionnaire, gender and age of participants, countries of birth, time spent in Germany, school education, religious affiliation, and health insurance status. Countries of birth and the gender proportion were compared to official data from population statistics. The local study coordinator forwarded this information to peer researchers on a weekly basis and encouraged peer researchers to reach out to the underrepresented groups.

After 4 weeks of field work, peer researchers, the respective partner organization, and the RKI met to evaluate the recruitment process. Peer researchers were encouraged to exchange experiences on recruitment strategies and support each other if they faced any difficulties. Furthermore, sociodemographic characteristics of the recruited study population were presented to discuss which groups might be underrepresented and to develop strategies on how to recruit these groups. After conclusion of data collection, a final meeting was scheduled with peer researchers, partner organization, and the RKI. Peer researchers received a certificate for their work as peer researchers, their participation in the training sessions, and the data collection. In addition, peer researchers, the local study coordinator, and RKI staff discussed experiences during training and fieldwork as well as the reception of the study within the MisSA community. Every peer researcher received a unique identifier, either an acronym or a number, to mark questionnaires of participants recruited by him- or herself. Peer researchers were compensated with €20 per questionnaire. Peer researchers were paid for 20 (30 in some cities) completed questionnaires only, even if they recruited more participants.

#### Data From Local HIV, Hepatitis B and C, and Sexually Transmitted Infection Testing Sites

**Figure 2:**
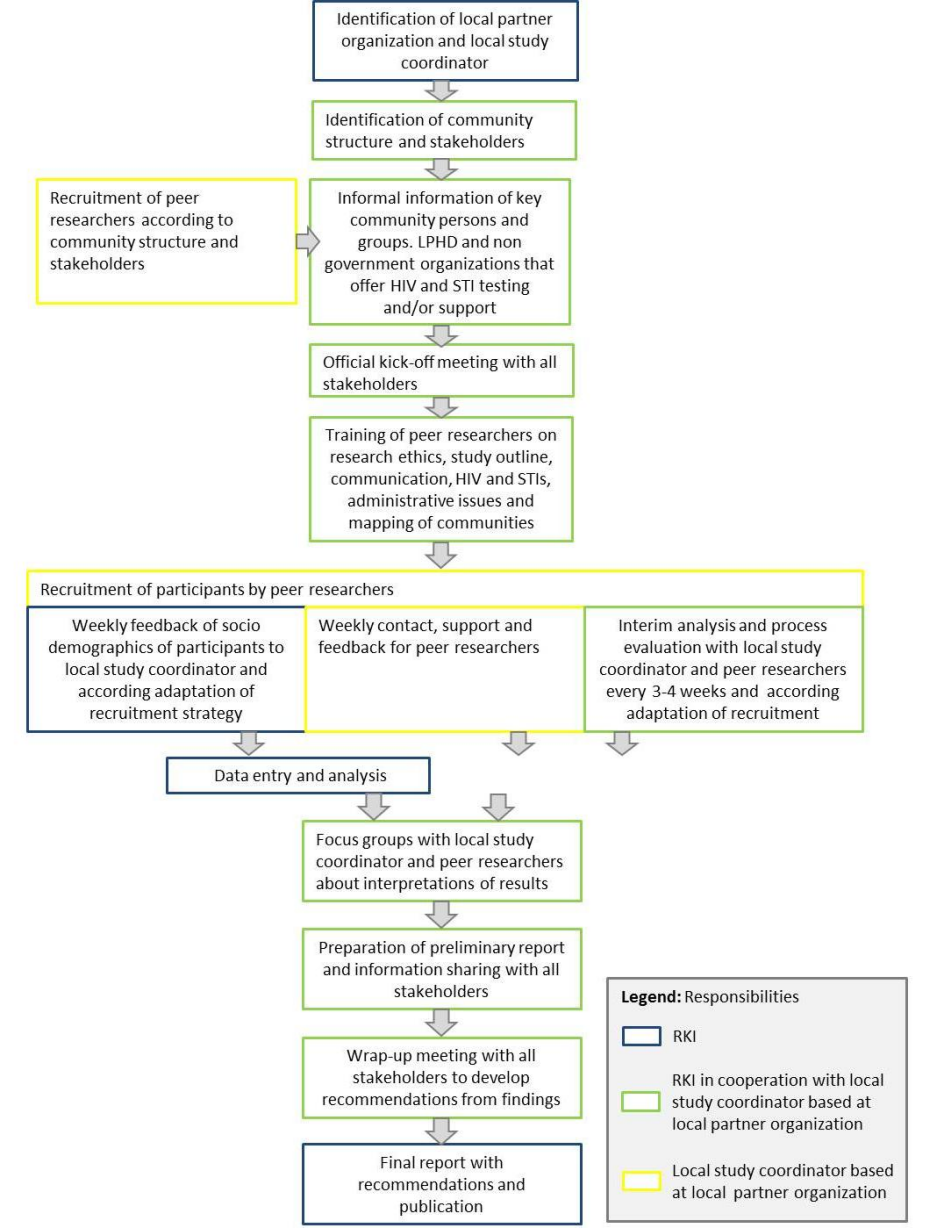
Overview of the study flow and assigned responsibilities. LPHD: local public health department; STI: sexually transmitted infection; RKI: Robert Koch Institute.

With completion of the questionnaire, participants were informed about the free and anonymous HIV, HEP, and STI testing service at the LPHD. We also included a flyer in English, German, or French with opening hours and language availabilities for counseling. For 6 months before data collection and 6 months after, the LPHD was sending the aggregated monthly numbers of MisSA attending the LPHD to assess whether the conduction of the study was leading to an increase of contacts and testing uptake among MisSA communities. Some LPHD agreed to send supplement aggregated information on countries of origin of their MisSA clients. Thus, it was possible to compare that data to population statistics to find out which groups were well reached or poorly reached. The study flow is shown in Figure 2.

### Data Entry and Statistical Analysis

Upon arrival at the RKI, a consecutive unique identifier was assigned to all questionnaires. Administrative data (eg, ID of peer researcher and mode of administration) and selected sociodemographic data were entered into an Excel database. During that process, questionnaires were inspected for completeness, consistency, and meeting of inclusion criteria. RKI staff entered data using Voxco Interviewer Web, an online survey and data collection software. A standard operation procedure for data entry and training was given to data entry personnel. A second person checked for correctness of entered data. We applied plausibility checks and excluded nonvalid cases from data analysis. Exclusion criteria were as follows: (1) missing data on country of birth and/or country of birth of parents, (2) missing data on sex, (3) participant not from sub-Saharan Africa or parents not originating from sub-Saharan Africa, (4) participant not living in or near study city, and (5) more than 40% of information missing.

Frequency distributions were tabulated for all variables, performing range checks and cross-validations. We described the characteristics of the study population in detail, for the whole sample, but also for each study city separately. We conducted stratified analysis of sociodemographic data and knowledge on HIV, HEP, and STIs; risk and protective behavior and attitudes toward people living with HIV and calculated unadjusted odds ratios. To identify subgroups with specific prevention needs, multivariable logistic regression was used to explore the associations between sociodemographic data and knowledge on HIV, HEP, and STIs; risk; and protective behavior and attitudes toward people living with HIV.

Per study city, we conducted a descriptive analysis of data directly after fieldwork and data entry. Variables on sociodemographics and sexual behavior, as well as potential risk factors, were stratified by gender; means, medians, and ranges were calculated where applicable. Variables on testing behavior and knowledge on HIV, HEP, and STIs were dichotomized to describe, in univariable analysis, differences in the following: gender, age (≤ 30 vs >30 years of age), educational level (no certificate and primary/secondary school vs high school/vocational school and university/college), time spent in Germany (< 5 years vs ≥ 5 years including *since birth*), German language proficiency (mother tongue, very good, good vs average, little, not at all), monthly net income (<€1000 vs ≥€1000), health insurance status (health insurance card vs medical treatment voucher, no health insurance at all, and unsure), religious affiliation (Christianity vs Islam), partnership (yes vs no), and mode of administration (interview vs self-completion). Odds ratios and 95% confidence intervals were calculated.

Data submitted by the LPHD was analyzed descriptively. All analyses were performed using the statistical software SPSS Statistics for Windows, version 20 (IBM Corp); for the whole sample, Stata, version 14 (StataCorp LLC) was used as well.

### Focus Group Discussions With Peer Researchers

Following data analysis for the respective study city, we conducted a focus group discussion with peer researchers, partner organization, and the RKI. Participants interpreted the results of descriptive analysis and discussed ideas for further data analysis. In addition, they identified the most important prevention messages and determined appropriate strategies and interventions to disseminate results into the MisSA communities. Peer researchers were thus involved in data collection and in the interpretation of survey results. Following the focus groups, we prepared a report for each study city including basic results of analysis and focus groups in order to disseminate these results to local organizations working with MisSA in HIV, HEP, and STI prevention; political decision makers; and to the MisSA community itself.

## Results

Data collection was completed in November 2016 when the last meeting with community partners and other stakeholders was conducted to discuss results and develop recommendations. Currently, data analysis is being conducted and we expect to publish first results by the end of 2017.

## Discussion

### Principal Findings

This study design was developed as a CBPHR project and people from different MisSA communities were involved in all stages of the research process. The active inclusion of research subjects results in higher acceptance of the research project in the target community and ultimately leads to increased accessibility and better quality of collected data [34,47,49,50]. Furthermore, the participation of MisSA in the development of study design and data collection assures a better understanding of the interests, needs, and living conditions of this group. Four of the MisSA who were part of the expert group became study coordinators in the local study cities and were thus involved in the complete research process. We chose to work with peer researchers for data collection to reach all relevant subgroups [37], including MisSA without (clear) legal status or people who might have faced discriminatory behavior by legal authorities in the past. Being able to reach “the unseen” with research is a major advantage of CBPHR [34,51]. We also included peer researchers in the discussion and interpretation of results as well in the development of recommendations to ensure relevance of findings for the local communities.

With the pilot study in Hamburg, we showed feasibility of study design for behavioral surveillance with MisSA. Peer researchers recruited 649 study participants within 2 months and overexceeded our aimed sample size of 350 MisSA. Working with peer researchers enabled us to reach particularly vulnerable groups, such as persons without health insurance (22% of participants) [44,45].

The described study design aimed to involve different stakeholders in the local study cities to establish new networks or expand existing collaborations for prevention planning and dissemination of results [34]. Thus, the utilization of CBPHR did not only allow better access to marginalized groups, but enabled us to get an enhanced understanding of the particular needs of the group under study and to develop research questions that were relevant to MisSA, stakeholders, and researchers. In addition, it will ultimately lead to concrete prevention messages for the group under study.

By providing true statements to assess knowledge on HIV, HEP, and STIs, participants can learn new information on these infections during questionnaire completion or interview. This is particularly helpful when reaching vulnerable subgroups that so far do not have access to prevention activities. When participants have questions about transmission or testing, peer researchers will refer them to either the partner organization or the LPHD. Gaining information about transmission and prevention of HIV, HEP, and STIs during survey completion, and being referred to counseling in the case of knowledge gaps, can be considered a preventive intervention.

### Limitations and Challenges

We used convenience sampling and, thus, cannot be sure that our sample will be representative for the MisSA population in Germany. Even though weekly feedback of sociodemographic data and subsequent adaptation of recruitment strategies were in place to steer sampling based on population statistics and to limit the effects of convenience sampling, these statistics did not include MisSA without legal status or MisSA who acquired German citizenship. When developing the study design, we had discussions with community partners who agreed that convenience sampling with peer researchers was the most feasible recruitment strategy to reach the MisSA community as well as MisSA with different living conditions.

Due to the cross-sectional nature of this survey, we will not be able to deduce direct causal relationships between sociodemographic, knowledge, and behavioral factors or the impact of prevention programs on behavior change.

The utilization of true statements might lead to an overestimation of knowledge on these indicators. In this context, we considered it ethically more important to inform participants who might belong to vulnerable hard-to-reach subpopulations about HIV, HEP, and STIs than to get exact information on level of knowledge. Additionally, expert group members highlighted the fact that taking part in a survey that actually provides information might be particularly motivating.

There might be social desirability bias in the answers of the respondents, especially when reporting the degree of condom use or talking about sexual practices and sexual violence. In addition, mode of administration might influence responses and social desirability.

The questionnaire was only available in English, French, and German; thus, MisSA who did not speak or read any of these languages can only be reached with interviews and simultaneous translation by peer researchers.

## Abbreviations

CBPHR: community-based participatory health research
ECDC: European Centre for Disease Prevention and Control
FGM/C: female genital mutilation/cutting
HEP: hepatitis B and C
HET: heterosexual contacts
KABP: knowledge, attitude, behavior, and practice
LPHD: local public health department
MisSA: migrants from sub-Saharan Africa
PLWH: people living with HIV
RKI: Robert Koch Institute
STI: sexually transmitted infection
WHO: World Health Organization
